# Autism Is Associated With Interindividual Variations of Gray and White Matter Morphology

**DOI:** 10.1016/j.bpsc.2022.08.011

**Published:** 2022-09-06

**Authors:** Ting Mei, Natalie J. Forde, Dorothea L. Floris, Flavio Dell’Acqua, Richard Stones, Iva Ilioska, Sarah Durston, Carolin Moessnang, Tobias Banaschewski, Rosemary J. Holt, Simon Baron-Cohen, Annika Rausch, Eva Loth, Bethany Oakley, Tony Charman, Christine Ecker, Declan G.M. Murphy, the EU-AIMS LEAP group, Jan K. Buitelaar, Jumana Ahmad, Sara Ambrosino, Bonnie Auyeung, Tobias Banaschewski, Simon Baron-Cohen, Sarah Baumeister, Christian F. Beckmann, Sven Bölte, Thomas Bourgeron, Carsten Bours, Michael Brammer, Daniel Brandeis, Claudia Brogna, Yvette de Bruijn, Bhismadev Chakrabarti, Tony Charman, Ineke Cornelissen, Daisy Crawley, Flavio Dell’Acqua, Guillaume Dumas, Sarah Durston, Christine Ecker, Jessica Faulkner, Vincent Frouin, Pilar Garcés, David Goyard, Lindsay Ham, Hannah Hayward, Joerg Hipp, Rosemary Holt, Mark H. Johnson, Emily J.H. Jones, Prantik Kundu, Meng-Chuan Lai, Xavier Liogier d’Ardhuy, Michael V. Lombardo, Eva Loth, David J. Lythgoe, René Mandl, Andre Marquand, Luke Mason, Maarten Mennes, Andreas Meyer-Lindenberg, Carolin Moessnang, Nico Mueller, Declan G.M. Murphy, Bethany Oakley, Laurence O’Dwyer, Marianne Oldehinkel, Bob Oranje, Gahan Pandina, Antonio M. Persico, Annika Rausch, Barbara Ruggeri, Amber Ruigrok, Jessica Sabet, Roberto Sacco, Antonia San José Cáceres, Emily Simonoff, Will Spooren, Julian Tillmann, Roberto Toro, Heike Tost, Jack Waldman, Steve C.R. Williams, Caroline Wooldridge, Iva Ilioska, Ting Mei, Marcel P. Zwiers, Christian F. Beckmann, Alberto Llera, Jan K. Buitelaar

**Affiliations:** Department of Cognitive Neuroscience, Donders Institute for Brain, Cognition and Behaviour, Radboud University Nijmegen Medical Centre, Nijmegen, the Netherlands; Department of Cognitive Neuroscience, Donders Institute for Brain, Cognition and Behaviour, Radboud University Nijmegen Medical Centre, Nijmegen, the Netherlands; Methods of Plasticity Research, Department of Psychology, University of Zurich, Zurich, Switzerland; Department of Forensic and Neuro-developmental Sciences, Institute of Psychiatry, Psychology and Neuro-science, King’s College London, London, United Kingdom; Department of Cognitive Neuroscience, Donders Institute for Brain, Cognition and Behaviour, Radboud University Nijmegen Medical Centre, Nijmegen, the Netherlands; University Medical Center Utrecht, Utrecht, the Netherlands; Department of Psychiatry and Psychotherapy, Central Institute of Mental Health, Medical Faculty Mannheim, University of Heidelberg, Mannheim, Germany; Department of Applied Psychology, SRH University, Heidelberg, Germany; Department of Child and Adolescent Psychiatry, Central Institute of Mental Health, Medical Faculty Mannheim, University of Heidelberg, Mannheim, Germany; Autism Research Centre, Department of Psychiatry, University of Cambridge, Cambridge, United Kingdom; Department of Cognitive Neuroscience, Donders Institute for Brain, Cognition and Behaviour, Radboud University Nijmegen Medical Centre, Nijmegen, the Netherlands; Department of Forensic and Neuro-developmental Sciences, Institute of Psychiatry, Psychology and Neuro-science, King’s College London, London, United Kingdom; Department of Psychology, Institute of Psychiatry, Psychology and Neuroscience, King’s College London, London, United Kingdom; Department of Child and Adolescent Psychiatry, Psychosomatics and Psychotherapy, University Hospital Frankfurt, Goethe University, Frankfurt, Germany; Department of Forensic and Neuro-developmental Sciences, Institute of Psychiatry, Psychology and Neuro-science, King’s College London, London, United Kingdom; Department of Cognitive Neuroscience, Donders Institute for Brain, Cognition and Behaviour, Radboud University Nijmegen Medical Centre, Nijmegen, the Netherlands; Oxford Centre for Functional MRI of the Brain, University of Oxford, Oxford, United Kingdom; Department of Cognitive Neuroscience, Donders Institute for Brain, Cognition and Behaviour, Radboud University Nijmegen Medical Centre, Nijmegen, the Netherlands; Karakter Child and Adolescent Psychiatry University Centre, Nijmegen, the Netherlands

## Abstract

**Background:**

Although many studies have explored atypicalities in gray matter (GM) and white matter (WM) morphology of autism, most of them relied on unimodal analyses that did not benefit from the likelihood that different imaging modalities may reflect common neurobiology. We aimed to establish brain patterns of modalities that differentiate between individuals with and without autism and explore associations between these brain patterns and clinical measures in the autism group.

**Methods:**

We studied 183 individuals with autism and 157 nonautistic individuals (age range, 6–30 years) in a large, deeply phenotyped autism dataset (EU-AIMS LEAP [European Autism Interventions—A Multicentre Study for Developing New Medications Longitudinal European Autism Project]). Linked independent component analysis was used to link all participants’ GM volume and WM diffusion tensor images, and group comparisons of modality shared variances were examined. Subsequently, we performed univariate and multivariate brain-behavior correlation analyses to separately explore the relationships between brain patterns and clinical profiles.

**Results:**

One multimodal pattern was significantly related to autism. This pattern was primarily associated with GM volume in bilateral insula and frontal, precentral and postcentral, cingulate, and caudate areas and co-occurred with altered WM features in the superior longitudinal fasciculus. The brain-behavior correlation analyses showed a significant multivariate association primarily between brain patterns that involved variation of WM and symptoms of restricted and repetitive behavior in the autism group.

**Conclusions:**

Our findings demonstrate the assets of integrated analyses of GM and WM alterations to study the brain mechanisms that underpin autism and show that the complex clinical autism phenotype can be interpreted by brain covariation patterns that are spread across the brain involving both cortical and subcortical areas.

Autism spectrum disorder (autism) is a heterogeneous condition characterized by difficulties with social and communicative behaviors, repetitive, rigid behaviors, and altered sensory processes (1). In search of the brain basis of autism, the condition has been associated with multiple morphological differences in gray matter (GM) and white matter (WM) (2,3), as reported by magnetic resonance imaging (MRI) studies. However, previous studies have shown heterogeneous findings of the alterations in both cortical (e.g., cortical thickness, surface area, volume) and subcortical (e.g., volume) morphometry in multiple brain regions, making it difficult to define the neural correlates of autism (3–5). Additionally, voxelwise GM volume analyses revealed divergent results, for instance, in temporal areas in autism (6–8). Studies of WM microstructural associations in autism are similarly heterogeneous in their findings (2,9,10). One explanation for discrepant and heterogeneous findings is that the studies differ widely in data analytic strategy—i.e., these studies rely on unimodal analysis techniques that ignore the signal of interest probably present in more than one modality (11). Additionally, when integrated together, these modalities might provide additional analytical sensitivity.

This prompted research to move beyond unimodality and incorporate and connect data from different imaging modalities. For example, Cauda *et al*. (12) suggested that GM variation in autism is generally accompanied by WM variation; Ecker *et al*. (13) showed higher axial diffusivity (L1) in the WM fiber tracts originating and/or terminating in the GM clusters with increased local gyrification in adults with autism. Despite the progression away from unimodal approaches, in essence, these MRI studies that correlate GM and WM measures do so after separate unimodal statistical analyses. This likely has less sensitivity to assess the biological variance than fully integrating multimodal data analysis across participants.

It is assumed that a relatively high level of co-occurring neurobiology underlying different aspects of brain morphology is due to the complicated nature of autism. Therefore, efficient modeling of this potential shared variance would increase the chances to produce a more complete picture of autism in a specific perspective (i.e., brain morphology in our study). Here, we aimed to use an integrative multimodal approach, linked independent component analysis (LICA), to simultaneously incorporate several imaging modalities allowing the investigation of intersubject variability across modalities in one analysis (14,15), which enables the isolation of artifacts and may increase the sensitivity to correlate the remaining signals with variables of interest (14). So far, studies that highlight the underlying shared variance between modalities using LICA in autism remain scarce. Previous studies revealed case-control differences between adults with autism and typically developing individuals in linked patterns of voxel-based morphometry (VBM) and diffusion tensor imaging (DTI) measures in several brain regions (16,17). However, these studies focused exclusively on autistic adults without intellectual disability and comprised relatively small sample sizes (<100 individuals) (16,17). Autism is a highly diverse condition; we therefore investigated brain patterns in a broader, more representative autism sample, which might help better characterize brain patterns of autism, which is one of the aims of the current study. We hypothesized that the model analysis would reveal the autism-related regional correspondence between GM and WM or modality-specific effect.

In addition to identifying categorical group differences, dimensional analyses, i.e., analyses of continuous scores of autism symptoms, might capture more of the heterogeneity of autism compared with categorical diagnostic labels. Many studies have demonstrated the univariate connections between GM or WM patterns and the core symptoms of autism [e.g., (2,3)]. Nonetheless, the relationships between brain substrates and clinical phenotypes are potentially the outcome of integrative effects across multiple autism symptom domains and brain areas, and therefore the multidimensional associations between brain covariations and core symptoms of autism need to be clarified. We therefore performed univariate analyses to identify the one-to-one dimensional associations and additionally implemented a multivariate analysis using canonical correlation analysis (CCA) to learn the integrated associations (18). Similarly, we furthermore expected that CCA would help to elucidate the potential correlation between brain and behavior.

This study was designed to overcome the aforementioned limitations of previous work by applying LICA to the Longitudinal European Autism Project (LEAP) dataset (19) to link the sources of variance of voxelwise GM volume and WM diffusion tensor measures. The LEAP dataset provides a deeply phenotyped and comprehensively biologically assessed multisite sample of individuals with and without autism that allows relating the results of LICA to clinical characteristics of the participants. More specifically, we applied 1) a univariate approach to identify categorical group differences of linking brain patterns and subsequently their one-to-one relationships to continuous measures of autism symptoms and 2) a multivariate method (i.e., CCA) to further quantify the association between two sets of brain patterns and autistic traits in the autism group.

## Methods And Materials

### Participants

The participants were part of the EU-AIMS (European Autism Interventions—A Multicentre Study for Developing New Medications) and AIMS-2-TRIALS (Autism Innovative Medicine Studies-2-Trials) LEAP dataset, a large multicenter study aimed at identifying and validating biomarkers in autism (19,20). Individuals with autism were included based on an existing clinical diagnosis according to DSM-IV, DSM-IV-TR, DSM-5, or ICD-10. Each participant underwent clinical, cognitive, and MRI assessment at one of 6 collaborative centers. Readers are referred to earlier EU-AIMS LEAP publications (19,20) for further details on experimental design and clinical characterization. In the present study, diffusion-weighted imaging data at time point 1 were available from participants in only 3 centers. Therefore, participants were selected who had both T1-weighted and diffusion-weighted imaging data available from the following centers: Institute of Psychiatry, Psychology and Neuroscience, King’s College London, United Kingdom; Radboud University Medical Centre, Nijmegen, the Netherlands; and Central Institute of Mental Health, Mannheim, Germany (Supplement Section 1).

The final sample comprised 344 participants between 6 and 30 years of age, including 185 autistic individuals (133 male and 52 female, IQ ≥40) and 159 nonautistic individuals (99 male and 60 female, IQ ≥50). The demographic and clinical information of the final sample is summarized in Table 1. For detailed exclusion criteria, see Supplement Section 2.

### Clinical Measures

The Autism Diagnostic Interview–Revised (ADI-R) (21) and the Autism Diagnostic Observational Schedule, Second Edition (ADOS-2) (22) were used to measure the past (ever and previous 4–5 years) and current core autism symptom severities in social interaction, communication, and restricted repetitive behavior (RRB) domains. Specifically, the calibrated severity scores for subscales and total of the ADOS-2 were calculated to use in the following analyses (23,24). Additionally, we used several parent-reported scales to assess autism behaviors, including the Social Responsiveness Scale, Second Edition (25) capturing social-communication variations, the Repetitive Behavior Scale–Revised (26) identifying repetitive and rigid behaviors, and the Short Sensory Profile (SSP) (27) evaluating sensory processing variations. Concerning the potential effect of the co-occurrence of attention-deficit/hyperactivity disorder, anxiety, and depression, we separately included the scores from an attention-deficit/hyperactivity disorder rating scale based on DSM-5 (28) and anxiety and depression scores from the Development and Well-Being Assessment (29) as additional covariates in the post hoc analyses. There was a substantial amount of missing clinical data, which could greatly reduce the power of our analysis. To tackle the missing clinical data and fully harness the large LEAP sample size, we used imputed clinical data (30). The imputation procedure was developed by our colleagues who considered the potential nonrandomness of missing data, and therefore developed quantitative measures to assess the quality of the imputations, and finally imputed data adopting a nonparametric tree regression model embedded in an iterative round-robin iterative schedule (30). The details of missingness of the current sample are included in Supplement Section 3.

### MRI Data Acquisition

All participants were scanned on 3T MRI scanners. High-resolution structural T1-weighted images were acquired with full head coverage at 1.2-mm thickness with 1.1- × 1.1-mm^2^ in-plane resolution. Diffusion-weighted imaging scans were acquired using echo-planar imaging sequence at 2-mm thickness with 2.0- × 2.0-mm^2^ in-plane resolution. MRI data acquisition parameters are included in Supplement Section 4.

### Image Processing

#### GM Volume Estimation

Structural T1 images were preprocessed according to the CAT12 toolbox (https://neuro-jena.github.io/cat//) pipeline in SPM12 (https://www.fil.ion.ucl.ac.uk/spm/software/spm12/) to obtain VBM data, which is a spatially unbiased whole-brain approach extracting voxelwise GM volume estimations. T1-weighted images were automatically segmented into GM, WM, and cerebrospinal fluid and affine registered to the Montreal Neurological Institute (MNI) template. A high-dimensional, nonlinear diffeomorphic registration algorithm (DARTEL) (31) was used to generate a study-specific template from GM and WM tissue segments of all participants and then to normalize all segmented GM maps to MNI space with 2-mm isotropic resolution. All GM images were smoothed with a 4-mm full width at half maximum isotropic Gaussian kernel.

#### Diffusion Parameters

Diffusion-weighted images from all sites were preprocessed using the same pipeline. Denoising was performed using the Marchenko-Pastur principal component analysis method (32). Subsequently, Gibbs-ringing artifacts were removed according to Kellner *et al*. (33). FSL eddy was employed to correct the eddy current–induced distortions and subject motion (34). To improve the final quality of data and recover most of the motion artifacts, we used intra-volume slice motion correction (35). Quality control reports were then generated for each subject and each site (36).

Individual voxelwise fractional anisotropy (FA), mean diffusivity (MD), mode of anisotropy (MO), L1, and radial diffusivity (RA) maps were derived using dtifit in FSL (37). These 5 DTI features were selected on account of the different aspects of WM microstructure; for example, FA measures the degree of anisotropic movement of water molecules, and L1 represents the magnitude of the diffusion in the primary direction, which are related to myelin structure or myelination. FA images were processed using a tract-based spatial statistics pipeline including registration of all images to FMRIB58_FA standard space, skeletonization of the mean group WM, and projection of each individual’s data onto the skeleton (38). The mean skeleton image was thresholded at FA 0.2. Other DTI measures (MD, MO, L1, RA) were projected onto the FA skeleton using the tbss_non_FA option. All DTI data had 1-mm isotropic resolution when entering the following data fusion model. A full quality control report and additional preprocessing details of the GM and WM images are included in Supplement Section 2.

### Modalities Fusing Analysis

The shared inter-participant variations across 6 features (i.e., VBM, FA, MD, MO, L1, RA) were explored using LICA (11). LICA is able to factorize the multiple input modalities simultaneously into modalitywise independent components (ICs) while importantly constraining all decompositions to be linked through a shared participant-loading matrix, which describes the amount of contribution of each participant to a specific IC. In addition to the participant-loading matrix, this method provides, per IC, a vector reflecting the contribution (weight) of each modality and a spatial map per modality showing the extent of the spatial variation. All mathematical algorithms of LICA are detailed in Groves *et al*. (11). As the model order is recommended to be less than 25% of the sample size (11,14), 80-dimensional factorization was chosen to perform LICA. A multimodal index (39) (Supplement Section 5) was calculated to present the contribution uniformity of the modalities in each IC. This results in a scalar value where 0 would equate to 100% unimodal contribution and 1 would mean equal contributions from all modalities.

### Statistical Approach

The participant loadings characterize the interindividual variations of the unimodal/multimodal effects, and in the current study, they were used for the analyses of group differences between autistic and nonautistic individuals and for associations with behavioral measures. Results reported in the main text were performed using imputed data to maximize the statistical power. All analyses were replicated using the original nonimputed data.

#### Case-Control Difference

A generalized linear model (GLM) was used to examine group differences of the brain’s inter-participant variations in LICA outputs while controlling for age, sex, IQ, and scanner site. Multiple comparison (number of tests = 80) correction was implemented using false discovery rate (FDR) (*p* < .05) (40). In addition to considering the effects of co-occurring conditions on case-control difference, we separately investigated the age-by-group, IQ-by-group, sex-by-group, and site-by-group interactions and medication use effects on brain pattern(s) with the case-control difference.

#### Brain-Behavior Associations

Similarly, we used a GLM to explore the univariate associations between each IC and subscales of ADI-R and ADOS-2, Social Responsiveness Scale, Second Edition, Repetitive Behavior Scale–Revised, and SSP in the autism group while controlling for age, sex, IQ, and scanner site. We corrected for multiple comparisons (number of tests = 80 × number of [sub]scale[s]) with FDR (*p* < .05). Subsequently, we used one CCA (18) to better picture the overall association between all brain ICs and subscales of ADI-R and ADOS-2 and total scores of Social Responsiveness Scale, Second Edition, Repetitive Behavior Scale–Revised, and SSP in the autism group. CCA is a multivariate approach to simultaneously learn 2 sets of linear projections corresponding to the brain ICs and the behavioral profiles, which maximizes the correlation between 2 sets of variables at the participant level. In such maximized correlation, the evaluation of brain-behavior relationships is based on the respective contribution of each IC and each behavioral profile to the correlation, which can be measured by the loading of each variable (transformed from canonical coefficient) described previously (41). Additionally, the canonical variates are calculated respectively for the brain and the behavioral sets according to the product of the canonical coefficients and the original sets. In this study, we referred to each pair of canonical variates as CCA mode. Before entering the CCA model, age, sex, IQ, and scanning site were controlled for both brain and behavior profile sets using the Huh-Jhun residualization method (42,43). The statistical significance of CCA modes was assessed by a complete permutation inference algorithm proposed by Winkler *et al*. (43), where both brain and behavior data were permuted separately across all participants with 10,000 iterations. For multiple testing correction of each CCA mode, we used a stepwise cumulative maximum approach (*p* < .05) [see details in (43)]. We further tested the reliability of the CCA findings and the stability of each loading of the significant CCA mode(s) using a leave-one-subject-out approach.

## Results

### Group Effect of Brain Components

**Figure 1:**
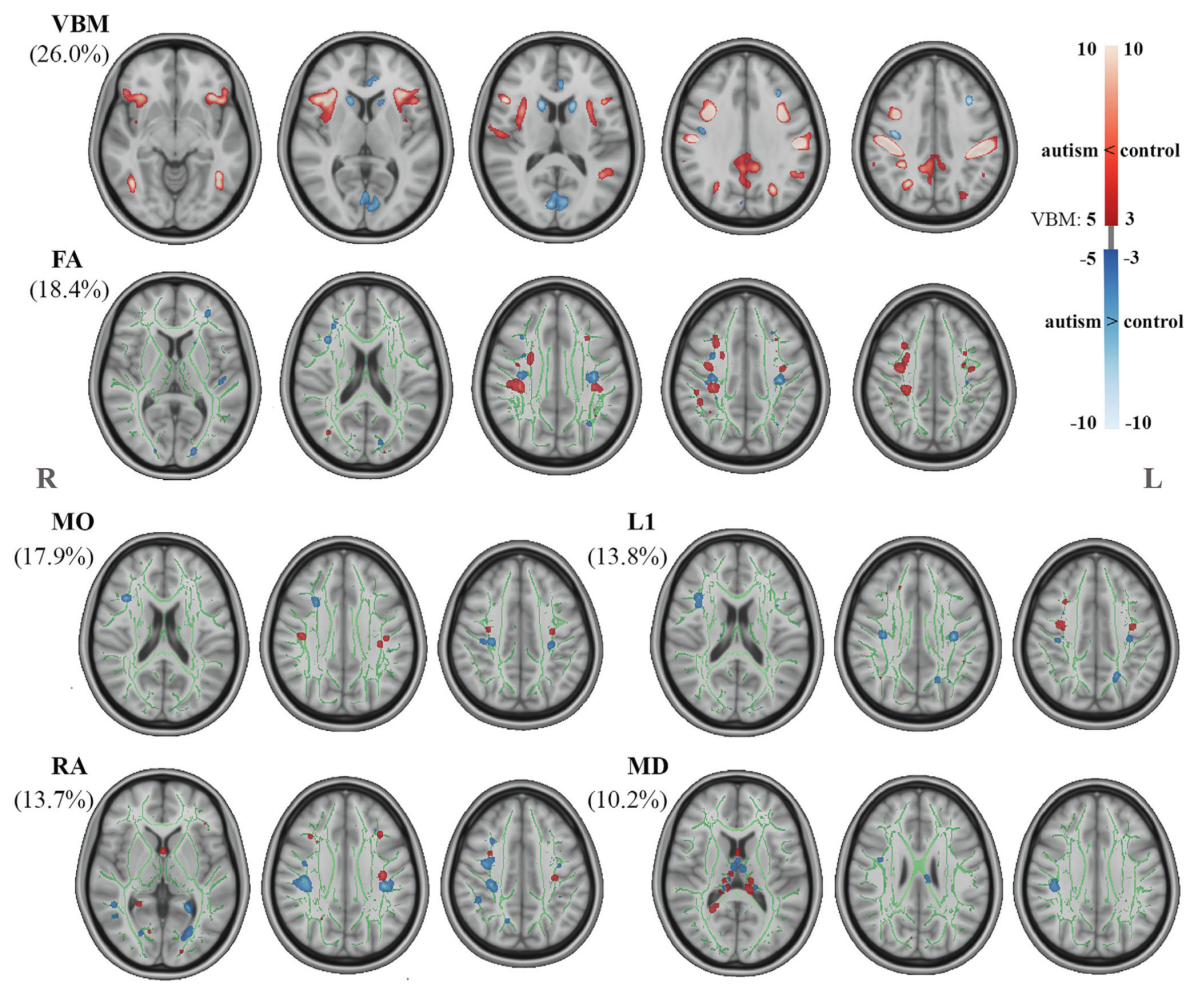
The multimodal component shows significant case-control difference. The relative contribution of each feature is displayed in parentheses. The voxel-based morphometry (VBM) spatial map is thresholded at 5<|*z*|<10. Clusters of diffusion tensor imaging features were filled and thresholded at 3<|*z*|<10, then smoothed using a 0.3-mm Gaussian kernel in FSL for visualization purposes. FA, fractional anisotropy; L, left; L1, axial diffusivity; MD, mean diffusivity; MO, mode of anisotropy; R, right; RA, radial diffusivity.

We obtained 80 ICs from the multimodal integration analysis in our study. The modality contributions (for 80 ICs) and multimodal index of each IC are in Supplement Section 5. We subsequently used the participant loadings of the 80 ICs to test for group differences and found one component (IC58) with a significant case-control difference (β = −0.192, *t*_337_ = −3.595, FDR-corrected *p* = .030) (Figure 1). The respective contributions of the modalities in IC58 are 26.0% from VBM, 18.4% from FA, 17.9% from MO, 13.8% from L1, 13.7% from RA, and 10.2% from MD, indicating that various MRI features share variance associated with autism. In Figure 1, we present the summarized images of each modality’s spatial map of IC58. The spatial patterns show autism-related smaller GM volume in the bilateral insula, inferior frontal gyrus, orbitofrontal cortex, precentral and postcentral gyrus, lateral occipital cortex, inferior temporal gyrus, angular gyrus, posterior division of cingulate gyrus, and precuneus cortex and larger GM volume in calcarine cortex, bilateral middle frontal gyrus, caudate, and anterior division of cingulate gyrus. Correspondingly, autism-related DTI features were found in bilateral superior longitudinal fasciculus (SLF), corticospinal tract (CST), and inferior fronto-occipital fasciculus (IFOF). In addition to these fasciculi, RA and MD in the cingulum and anterior thalamic radiation were also implicated. Taken together, SLF and their adjacent GM volumes (i.e., involved frontal, precentral, and postcentral areas [Supplement Section 6]) in autism indicate that variations of GM volumes and WM microstructure are linked in these brain locations, rather than dependent on modality or tissue.

Post hoc, to assess the respective influences of co-occurring conditions, diagnosis-by-age, diagnosis-by-sex, or diagnosis-by-IQ interactions, and medication use on the multimodal IC found significantly associated with group, we additionally included them as separate covariates in the GLM of IC58. The analysis showed that the group effect of IC58 was robust to the inclusion of these additional covariates in the model (*p* < .01). However, we found a significant moderate site-by-diagnostic group interaction effect on the current result (*G*^2^_2_ = 6.860, *p* = .032). This was driven by having significant effects in 2 of the 3 sites with no significant differences in the third. Details are in Supplement Section 7.

### Relating Brain Patterns to Behavior Profiles

**Figure 2:**
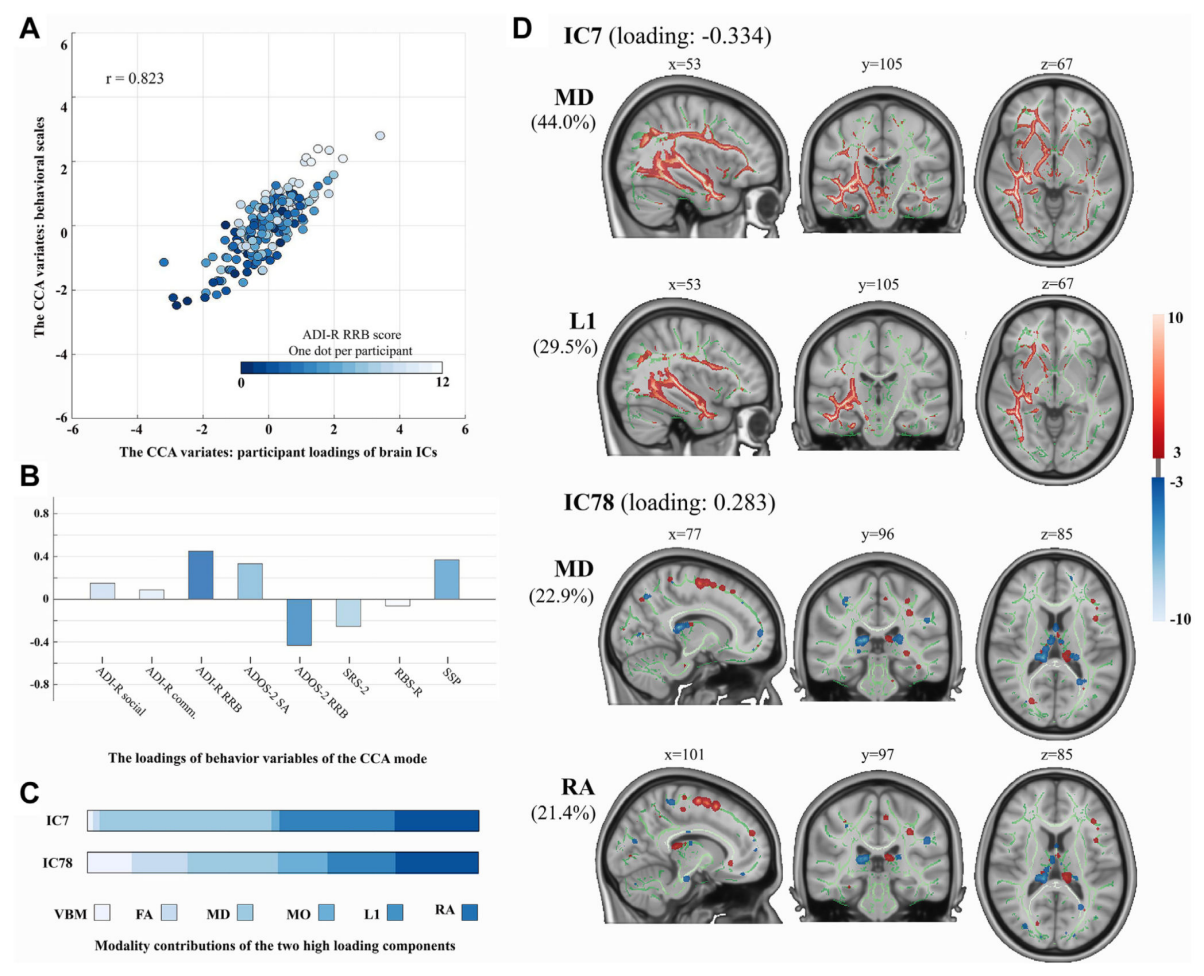
The multivariate association pattern (i.e., canonical correlation analysis [CCA] mode) was found to be significant between the 2 sets of brain components and all behavioral profiles. **(A)** The scatterplot of this correlation (between the CCA variates); x- and y-axes represent the pair of CCA variates. One dot per each participant is coded with gradient color relating to the restricted repetitive behavior (RRB) subscale of the Autism Diagnostic Interview-Revised (ADI-R). **(B)** The loadings of each behavioral (sub)scale in this CCA mode. **(C)** The modality contributions to the components displayed in panel **(D)**. **(D)** The 2 multimodal components with a strong contribution to the correlation with autism core symptoms; the top 2 loading modalities in each component are shown. The canonical loading of each component is shown in parentheses. The modality spatial maps are thresholded at 3<|*z*|<10. The CCA was performed only in the autism group. ADOS-2, Autism Diagnostic Observational Schedule, Second Edition; comm., communication; FA, fractional anisotropy; IC, independent component; L1, axial diffusivity; MD, mean diffusivity; MO, mode of anisotropy; RA, radial diffusivity; RBS-R, Repetitive Behavior Scale-Revised; SA, social affect; SRS-2, Social Responsiveness Scale, Second Edition; SSP, Short Sensory Profile; VBM, voxel-based morphometry.

We conducted the univariate (GLM) and multivariate (CCA) correlation analyses on brain and behavior data in the autism group only. No significant univariate brain-behavior relationship in the autism group was found (FDR-corrected *p* > .120). We did, however, find a significant multivariate association pattern of CCA (*r* = 0.823, corrected *p* = .006) (Figure 2). The proportion of total variance explained by this multivariate pattern was 20.8% for brain ICs and 14.2% for behaviors. In this multivariate associated pattern, multimodal IC7 (canonical loading: −0.334) and IC78 (canonical loading: 0.283) showed strong contributions to the correlation with autism core symptoms, and from a phenotypic perspective, this multivariate pattern demonstrated a strong association with the ADI-R RRB and ADOS-2 RRB subscales. WM microstructure mainly dominated in IC7 and IC78. IC7 mainly included right inferior longitudinal fasciculus, IFOF, and CST, and IC78 primarily involved bilateral anterior thalamic radiation and SLF. These two predominant ICs highlight the involvement of WM in autism symptoms. The loadings of each brain component of this CCA mode can be found in Supplement Section 8. The leave-one-subject-out analysis indicated that the significant CCA mode of CCA analysis was reliably estimated (Supplement Section 9). We additionally ran a CCA model excluding SSP to probe the effect that the imputed SSP scores (42%) specifically may be having and found the entire structure of the output did not differ greatly (Supplement Section 10).

The results using nonimputed data of group effect and univariate brain-behavior association were similar to the main results. The different CCA patterns using nonimputed data were reasonable owing to the large amount of missingness (Supplement Section 3).

## Discussion

We examined autism-related interindividual variance of integrated GM-WM morphology in a large European sample of individuals with and without autism across a broad age and IQ range. Analyses showed a significant diagnostic group effect of the linked GM-WM pattern that supports our hypothesis of the link between GM and WM morphology alterations in autistic individuals. In particular, the GM volume variation in precentral and postcentral areas converged with the WM microstructural variation in the SLF. This spotlights the shared variances between GM and WM morphology in these brain areas in autism and suggests that the structural associations in autism are not only limited to localized regions, but also involve the WM pathways connecting these brain areas. In a next set of analyses, we found a significant integrative association between brain patterns and autism core behaviors using CCA in the autism group, where the identified brain multimodal patterns underline the important role of WM morphology.

Notably, the autism-specific VBM pattern on this multimodal analysis replicates to a certain extent our previous unimodal GM volume covariation study in a larger overlapped sample of the EU-AIMS project (8). The areas of bilateral insula, inferior frontal gyrus, orbitofrontal cortex, and caudate form a steady autism-related covariation pattern in previous and current studies. These areas were demonstrated previously to relate to repetitive behaviors and reward-based decision-making abilities in autism (44,45). The covariation of insula and frontal areas in our studies indicates the consistency and stability of the co-occurring GM morphological alterations in autism. Benefiting from multimodal/multivariate approaches suggested by previous studies (46,47), the application of the LICA approach modeling the shared variances across modalities extends identified autism-related GM associations to precentral, postcentral, occipital, and temporal areas and additionally links with significant WM findings of DTI measures.

Our results indicated one covarying set of brain GM and WM areas associated with autism diagnosis. In this multimodal set, GM volume in cortical and subcortical regions and microstructure in WM tracts (mainly SLF, CST, and IFOF) were implicated, and these regions/tracts have previously been identified in unimodal analyses (10,44,48–50). This broad range of brain regions along with large WM bundles associated with autism is in accord with the notion that the neural correlates of autism are widespread in brain regions and connectivity patterns (51–53). This also corresponds with another multimodal autism study reporting extensive autism-related brain areas (16). The areas of this IC have been linked previously to both social and nonsocial cognitive difficulties in individuals with autism, varying from visual, sensory, and motor processing to high-order cognitive abilities (10,54–57). For example, precentral and postcentral gyrus, SLF, and CST are related to (sensory-)motor processing and have been implicated in autism (10,50,58). These adjacent affected areas (grouped areas of precentral and postcentral areas, SLF, and CST; grouped areas of lateral occipital cortex and IFOF occipital section) in our findings logically are in line with the brain organization principles during development, which states that nearby areas tend to be more interconnected (59,60). In summary, the autism diagnosis–related covarying GM-WM pattern reflects that autism is a complex condition associated with neural morphology. However, we did not find any significant univariate relationship between behavioral phenotypes and brain patterns. This is probably a result of the diverse phenotypes in our sample (i.e., complex and heterogeneous nature of autism); therefore, the compound variances of the symptom profiles cannot be explained by single unimodal/multimodal brain patterns. Additionally, imaging studies suggested that individuals with autism develop alternative processing strategies (52) that might mix or neutralize the manifestations of behavioral phenotypes in autism moderating detection of well-established brain-behavior relationships. Furthermore, nonsignificant univariate relationships but one remarkable multivariate brain-behavior relationship in the current study may relate to the relatively mild autism traits in the LEAP cohort; for example, the average total score of ADOS-2 calibrated severity scores is lower than the clinical cutoffs, which was reported in the larger LEAP sample compared with other cohorts (20).

The significant multivariate brain-behavior relationship in the current study is one prominent WM-dominated multivariate relationship between all brain patterns and all behavioral profiles. The top two ranking ICs emphasize the importance of WM connection to the core traits of autism. Multivariate/multimodal analysis increases the difficulty in interpreting findings, as it is challenging to clarify the direction of each association. Nonetheless, coinciding with previous studies (2,10,50,61,62), there are associations of inferior longitudinal fasciculus, IFOF, CST, and SLF microstructural measures with core symptoms/traits in autism. In line with previous findings, our work also shows laterality effects with much of the contribution from IC7 being right lateralized in autism (55,63). Significantly, GM volume contributed only a small amount, which indicates that WM morphology has a stronger connection to the autism behavioral phenotypes compared with GM in this multivariate correlation. In our previous GM work, a multivariate correlation pattern exhibited a strong association between RRB scores of ADI-R and ADOS-2 and GM covariations in autism, while here, when including WM microstructural measures, the brain patterns demonstrated a strong association with RRB domains of the ADI-R and ADOS-2. This multivariate brain-behavior association needs further investigation to determine the relationship between the development of WM microstructure and behaviors, to determine the generalizability beyond the current sample, and to explore how different behavioral scales capture behavioral phenotypes in autism, which might expand our knowledge of current brain-behavior association patterns.

Our findings should be interpreted with regard to several limitations. First, to generalize our pattern of brain alterations associated with autism requires replication in other large-scale datasets. Second, the current multimodal dataset included fewer participants than our previous work (8), which may have lowered statistical power when detecting the group effects and brain-behavior associations in autism group. Despite that, this is still the largest multimodal MRI study of autism to date and includes a diverse sample of autistic and nonautistic participants. Third, limited to the cross-sectional nature of the current study, our findings are deficient to address the developmental effects on these brain patterns and their relations to the behavior profiles, as the structures of the brain (especially WM) change remarkably over puberty and with aging.

In this study, we demonstrated autism-related interindividual covariations of GM volume in frontal, precentral, postcentral, and occipital areas and microstructure in associated WM fasciculi. Together, these GM and WM alterations are part of the underlying neural substrates of the phenotypes in autism. Subsequently, we highlighted the potential role of WM in relation to the core symptoms of autism. Further studies may link our GM-WM morphometric findings with brain function acquired from cognitive assessments and/or functional MRI data.

## Supplementary Material

Supplementary material cited in this article is available online at https://doi.org/10.1016/j.bpsc.2022.08.011.

Supplementary Material

## Figures and Tables

**Table 1:** Demographic and Clinical Information of Participants^a^

	Autism Group, *n* = 185	Control Group, *n* = 159	*t*/χ^2^	*p* Value
Demographic Information				
Age, Years^b^	17.30 (5.22)	17.51 (5.19)	0.369	.712
IQ*^b,c^*	98.90 (20.44)	102.6 (19.10)	1.769	.079
IQ ≥ 75	105.47 (15.30)	107.32 (14.04)	1.083	.028
IQ < 75	66.28 (6.95)	63.88 (8.55)	–0.994	.329
IQ ≥ 75/ IQ < 75^d^	154 (83.2%)/ 31 (16.8%)	142 (89.3%)/ 17 (10.7%)	2.620	.106
Sex, Female/ Male^d^	52 (28.1%)/ 133 (71.9%)	60 (37.7%)/ 99 (62.3%)	3.610	.057
Clinical Profiles				
ADI-R				
Social interaction	16.54 (6.95)			
Communication	13.35 (5.57)			
RRB	4.07 (2.58)			
ADOS-2 CSSs				
Total	5.40 (2.75)			
Social affect	6.06 (2.64)			
RRB	4.70 (2.77)			
SRS-2 Raw	70.80 (11.55)	55.78 (11.89)		
Score^e^				
RBS-R^e^	15.54 (13.54)	5.30 (6.05)		
SSP^e^	142.16 (23.63)	166.66 (17.76)		

Values are mean (SD) or *n* (%).

ADI-R, Autism Diagnostic Interview–Revised; ADOS-2, Autism Diagnostic Observational Schedule, Second Edition; CSSs, calibrated severity scores; RBS-R, Repetitive Behavior Scale–Revised; RRB, restricted repetitive behavior; SRS-2, Social Responsiveness Scale, Second Edition; SSP, Short Sensory Profile.

a IQ and symptom profiles reported are imputed data (30).

b Statistical differences were assessed by two-sample *t* test. Degrees of freedom of the two *t* tests were 342.

c IQ ranged from 40 to 148 in the autism group and from 50 to 142 in the control group.

d The differences were examined by χ2 test.

e In SRS-2, RBS-R, and SSP questionnaires, we used parent-rated report.
