# Supplementary material for: Autism Is Associated With Interindividual Variations of Gray and White Matter Morphology
Source: Biol Psychiatry Cogn Neurosci Neuroimaging. Author manuscript; Available in PMC 2026 Mar 28. (PMC7618945; doi:10.1016/j.bpsc.2022.08.011)
Supplement: Supplementary Material [file EMS212886-supplement-Supplementary_Material.pdf]

SUPPLEMENTARY INFORMATION

Autism Is Associated With Interindividual Variations of Gray and White Matter Morphology

Contents

1. **Demographic information of each site and comparison of current and previous sample** ... 2
2. **Quality control report** ... 5
3. **Missingness of the original data and statistical analyses** ... 7
4. **MRI data acquisition parameters** ... 11
5. **Modality contributions and Multimodal Index (MMI)** ... 12
6. **Spatial distribution of VBM and DTI measures in the component with significant group effect** ... 13
7. **Sensitivity Analyses** ... 14
8. **All brain IC loadings of the significant CCA mode** ... 21
9. **Reliability and stability assessment of CCA results** ... 22
10. **Probing potential effect of the imputed SSP scores on CCA outputs** ... 26
11. **Additional exploratory analyses with respect to IQ** ... 29

Supplementary References ... 30

1. Demographic information of each site and comparison of current and previous sample

Table S1. Demographic information of participants in each site

	KCL		Nijmegen		Mannheim	
Variable	Autism	Control	Autism	Control	Autism	Control
N	91	71	72	55	22	33
Age, mean (SD)	17.87 (5.24)	16.69 (5.84)	16.95 (5.62)	15.92 (4.08)	16.07 (2.86)	15.46 (3.03)
IQ, mean (SD)	99.04 (22.19)	104.48 (22.70)	97.87 (19.15)	97.14 (15.29)	101.73 (15.71)	108.02 (12.71)
Sex, N (%)						
Male	64 (70.33)	40 (56.34)	52 (72.22)	36 (65.45)	17 (77.27)	23 (69.70)

SD, standard deviation; IQ, full-scale intelligence quotient.

To address the concern of inclusion bias, we compared the distributions of the demographic data and symptom severity between the sample in the current study (non-imputed data were used) and the sample without diffusion data that was included in the original sample (1). The statistical outputs are summarized in Table S2. The results demonstrated the control group in the current sample is significantly older than the excluded previous sample in average, and this likely relates to the effects of site.

Table S2. Comparison of descriptive of the current sample and the sample without diffusion data in the previous paper (1)

	Autism			Controls		
	Current Sample	Excluded Previous Sample[a]	Statistics	Current Sample	Excluded Previous Sample[a]	Statistics
Diagnosis (N)	185	174		159	100	
Age (Mean, SD)	17.30, 5.22	16.20, 5.80	t(357)=1.88, p=0.061	17.51, 5.19	15.81, 6.39	t(179)=2.23, p=0.027
IQ (Mean, SD)[b]	99.01, 20.51	98.70, 18.05	t(355)=0.15, p=0.879	102.68, 19.22	107.12, 17.56	t(255)=-1.87, p=0.063
Sex (Male, Female)	132, 52	130, 44	χ^2=0.40, p=0.526	99, 60	69, 31	χ^2=1.22, p=0.269
ADI (Mean, SD)[c]						
Social Interaction	16.48, 7.03	17.46, 6.31	t(342)=-1.36,p=0.176			
Communication	13.31, 5.63	13.88, 5.61	t(342)=-0.93, p=0.351			
RRB	4.07, 2.61	4.63, 2.70	t(342)=-1.96, p=0.051			
ADOS CSS (Mean, SD)[d]						
Total	5.41, 2.77	5.29, 2.75	t(349)=0.41, p=0.682			
Social Affect	6.04, 2.66	5.99, 2.54	t(349)=0.18, p=0.857			
RRB	4.73, 2.78	4.70, 2.77	t(349)=0.12, p=0.907			

[a] The sample without diffusion data was included in (1).

[b] In non-imputed data of the current sample, there were 2 participants in autism and 2 participants in the control group missing IQ.

[c] There were 5 participants without ADI scores in the current sample, and 10 participants without ADI scores in the previous sample here.

[d] There were 4 participants without ADOS scores in the current sample, and 4 participants without ADOS scores in the previous sample here.

SD, standard deviation; IQ, full-scale intelligence quotient; ADI, Autism Diagnostic Interview-Revised; RRB, restricted, repetitive behaviors; ADOS, Autism Diagnostic Observational Schedule 2; CSS, calibrated severity scores; SA, social affect.

2. Quality control report

The current work preceded our previous work (1), where we used 604 participants with preprocessed voxel-based morphometry (VBM) data. In the previous work we excluded those without FIQ (n=5) however here we use imputed demographic and clinical data and therefore include those participants. New participants were additionally added (N=96). This gave us 700 subjects in total with VBM data. However, in our LEAP wave 1 dataset, only 3 scanning sites had diffusion weighted imaging (DWI) data of appropriate quality limiting our total sample to 418 participants.

Quality control reports of preprocessing of DWI data were generated for each participant and each site (three sites included: KCL, Nijmegen and Mannheim) (2). 49 participants with excessive motion during acquisition (absolute motion > 4 mm translation), with high number of outliers (>6%) were excluded from the study. To also correct for possible signal drop-out in the b0 data, b0 voxels for each slice were compared and outliers were identified using the conventional interquartile range (IQR) method for outlier detection (outlier threshold=3rd quartile+1.5xIQR). Slices that were considered outliers were then replaced with the corresponding median b0. Finally, to make the diffusion data more uniform across sites, all data was resampled using a real symmetric spherical harmonics (SH) representation of the DWI signal limited to SH degrees l=6. This allowed a further reduction of high frequency noise and allow the detection and replacement of remaining data outliers not fully recovered by eddy. Because some of the datasets from Siemen's scanners still exhibited residual Gibbs ringing in b0s data compared to the other sites, a final Gibbs ringing correction was applied to all datasets using ExploreDTI only to b0 data (3). All data was visually inspected to ensure that the final dataset was free from any unexpected artifact or problem not detected by the automatic pipeline. This included the search for brain volumes not correctly planned (i.e., incomplete volume acquisition), bad brain masking and MRI issues like radio frequency (RF) spikes and coil failures. In total, 18 subjects were excluded from further analysis. After visual inspection data quality looked consistent across datasets and with no visible residual artifacts. Please note, because Nijmegen

and Mannheim exhibited a high Rician noise floor in the raw data (4), before running the main pre-processing pipeline, datasets from these sites were pre-corrected using an in-house implementation of the methods described in (5) to reduce the bias in the DWI signal induced by the noise floor. The above procedures resulted in 351 participants in total with high quality DWI data, in which 3 participants without T1 images were excluded, therefore there were 348 participants entering VBM and DTI generations.

A VBM quality control report was generated (based on the participants with both T1 and DWI images) by the CAT SPM pipeline for each participant that included visual evaluation of the segmentation, and quantitative quality measures including mean correlation from sample homogeneity module and weighted overall image quality ratings that were additionally used to detect and exclude images of insufficient quality for inclusion in analysis. Accordingly, 1 participant failed to be segmented and was excluded (as uncorrected white matter hyperintensities were kept as GM after segmentation). Then we visually checked the images with the mean correlation smaller than three standard deviations (SDs) from the sample mean and with the weighted overall image quality rating larger than three SDs from the mean, which led to a visual inspection of 8 participants after preprocessing, and subsequently 2 of 8 participants were excluded as the T1 images of these two participants were observed obvious artifacts due to head motion, and after segmentation there were strong noises observed of their GM images and low weighted average image qualities were rated for the segmentation..

Subsequently, we checked diffusion tensor imaging (DTI) measures using sum of squared error (sse) of diffusion tensor fit that were generated for each participant by FSL DTIFIT. We visually checked the sse images, and excluded 1 participant due to an obvious artifact across the brain. This low number is due to previous exclusion during preprocessing.

In the end, there were 344 participants included in our final analyses.

3. Missingness of the original data and statistical analyses

Table S3. Missingness of clinical and demographic measures in current sample

clinical/demographic measure	Subscales	Autism, N=185		Controls, N=159	
		Observations (N)	missing (N)	Observations (N)	missing (N)
Diagnosis	/	185	0	159	0
age	/	185	0	159	0
sex	/	185	0	159	0
IQ	/	183	2	157	2
ADHD rating scale (parent-report)	Inattentiveness	158	27	88[a]	71
	Hyperactivity/Impulsivity	158	27	88[a]	71
ADI	Social Interaction	180	5	/	/
	Communication	180	5	/	/
	RRB	180	5	/	/
ADOS	CSS Total	181	4	/	/
	Social Affect CSS	181	4	/	/
	RRB CSS	181	4	/	/
SRS (parent-report)[b]	Total	154	31	90[a]	69
RBS (parent-report)[b]	Total	157	28	90[c]	69
SSP (parent-report)[b]	Total	108	77	72[c]	87
DAWBA	Anxiety	158	27	127	32
	Depression	142	43	112	47
Medication[d]	/	176	9	150	9

[a] In adult TD group (IQ≥75), only self-reported questionnaires were administered.

[b] The correlations between brain and behavior profiles were only performed in autism group. Therefore, the percentages of missingness were computed according to autism group.

[c] The questionnaires were not administered in the adult TD group (IQ≥75).

[d] Medication data were not imputed.

Case-control difference

Due to 4 individuals lack of full-scale IQ (IQ), a generalized linear model (GLM) was utilized to examine group difference of the brain's inter-participant variations in linked independent component analysis (LICA) outputs on the 340 participants while regressing out the effect of age, sex, IQ and scanner site.

We found the same multimodal component (IC58) significant relating to autism (β=-0.189, t(333)=-3.514, FDR corrected p=0.040) as using imputed data.

Brain-symptom associations

Univariate analyses

We used a GLM to explore the univariate associations between each independent component (IC) and subscales of Autism Diagnostic Interview-Revise (ADI) (6) and Autism Diagnostic Observational Schedule 2 (ADOS) (7), Social Responsiveness Scale 2nd Edition (SRS) (8), Repetitive Behavior Scale-Revised (RBS) (9), and Short Sensory Profile (SSP) (10) in the autism group correcting for multiple comparison with false discovery rate (FDR) ($p<0.05$).

There are 178 individuals with ADI scores (5 individuals without ADI scores), 179 individuals with ADOS scores (4 individuals missing ADOS scores), 154 individuals with SRS score (31 missing), 157 individuals with RBS score (28 missing) and 108 individuals with SSP score (77 missing) in autism group. Similar to the main findings with imputed data, we did not find any univariate brain-symptom association (FDR corrected $p>0.05$).

Multivariate analyses

Subsequently, we utilized canonical correlation analysis (CCA) (11) to detect multivariate association between all brain ICs and all symptom phenotypes in the autism group. The statistical significance of CCA modes was assessed by permutation inference (12). For multiple testing correction, we used stepwise cumulative maximum approach, $p<0.05$. The evaluation of the contribution of each IC and each behavioral measure to the canonical correlation was according to the loading of each variable described previously (13).

There were 105 of 185 participants involved in the CCA due to the missing data of the behavioral profiles, which drew a different picture of CCA output compared to the analysis using imputed data ($r=0.973$, corrected $p=4.000\times10^{-4}$, Figure S1). In this multivariate correlation pattern, IC29 (canonical

loading: 0.291) and IC79 (canonical loading: -0.287) demonstrated a strong contribution to the correlation. Meanwhile, ADI social, RBS and ADOS RRB demonstrated a strong contribution to the correlation . The different multivariate correlation patterns of non-imputed and imputed data are the reasonable outcome owing to the probable changes of structure and variances in the data. Additionally, the less sample size and lots of input features might lead to unstable results of CCA.

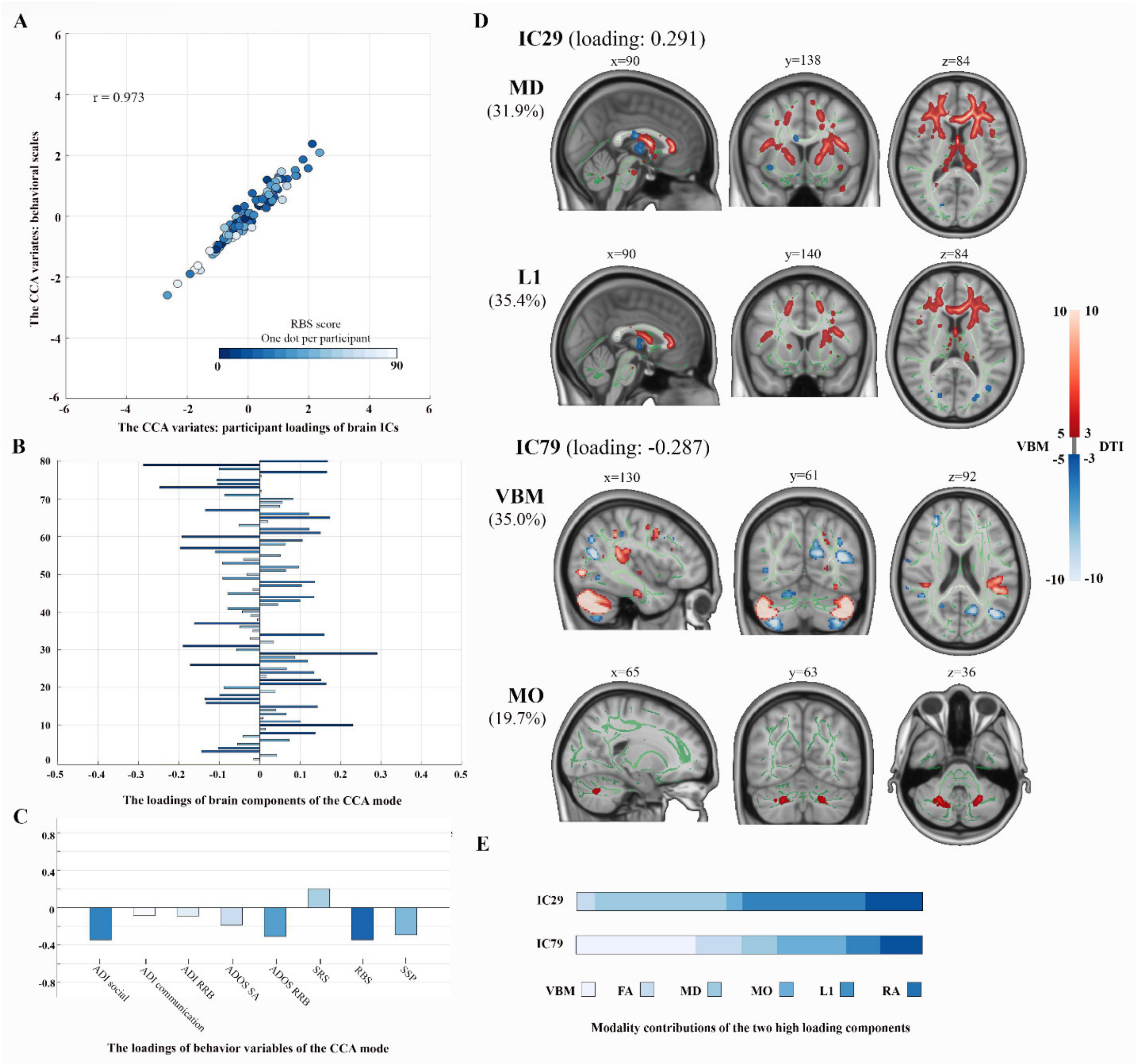

Figure S1. The multivariate association pattern (i.e., CCA mode) was found significant between the two sets of brain components and all behavioral profiles using non-imputed data (N=105). A displays the

scatterplot of this correlation (between the CCA mode), and x, y axes are the pair of CCA variates. One dot in each participant is coded with gradient color regarding to the total score of RBS. B demonstrates the loading of each brain component in this CCA mode. C demonstrates the loading of each behavioral variable in this CCA mode. D exhibits the two brain components with the strong contribution to the correlation with autism core symptom, where the top two loading modalities in each component are shown in the figure. The canonical loading of each component is shown in the brackets. The VBM spatial map is thresholded at $5<|Z|<10$. The spatial maps of DTI features were filled and thresholded at $3<|Z|<10$. E shows the modality contributions to the brain components displayed in D. The CCA was only performed in autism group. CCA, canonical correlation analysis; ADI, Autism Diagnostic Interview-Revised; ADOS, Autism Diagnostic Observational Schedule 2; SA, social affect; RRB, restricted repetitive behavior; SRS, Social Responsiveness Scale 2nd Edition; RBS, Repetitive Behavior Scale-Revised; SSP, Short Sensory Profile; IC, independent component; MO, mode of anisotropy; RA, radial diffusivity; MD, mean diffusivity; FA, fractional anisotropy; L1, axial diffusivity; VBM, voxel-based morphometry.

4. MRI data acquisition parameters

Table S4. Summary of acquisition parameters across sites

	KCL	Nijmegen	Mannheim
Manufacturer	GE Medical systems	Siemens	Siemens
Model	Discovery mr750	Skyra	TimTrio
Software Version	LX MR DV23.1_V02_1317.c	Syngo MRD13	Syngo MR B17
**T1-weighted image**			
Acquisition sequence	SAG ADNI GO ACC SPG	Tfl3d1_16ns	MPRAGE ADNI
Coverage	256*256	256*256	256*256
slices	196	176	176
Thickness [mm]	1.2	1.2	1.2
Resolution [mm^3]	1.1*1.1*1.2	1.1*1.1*1.2	1.1*1.1*1.2
TR [s]	7.31	2.3	2.3
TE [ms]	3.02	2.93	2.93
FA [°]	11	9	9
FOV	270	270	270
**EPI diffusion weighted sequence**			
TR / TE (ms)	12000 / 67	12000 / 103	12000 / 102
Flip angle (°)	90	90	90
Slice thickness (mm) / No.	2 / 72	2 / 72	2 / 72
In-plane resolution (mm2)	2 x 2	2 x 2	2 x 2
B-values (s/mm2)	0 / 1500	0 / 1500	0 / 1500
No. of gradients	6 / 60	6 / 60	6 / 60

5. Modality contributions and Multimodal Index (MMI)

MMI(14):

$$MMI(w)=1-N*((max(w)-1/n)/n-1) \qquad \text{equation S1}$$

w=modality contributions in each component, n= number of modalities.

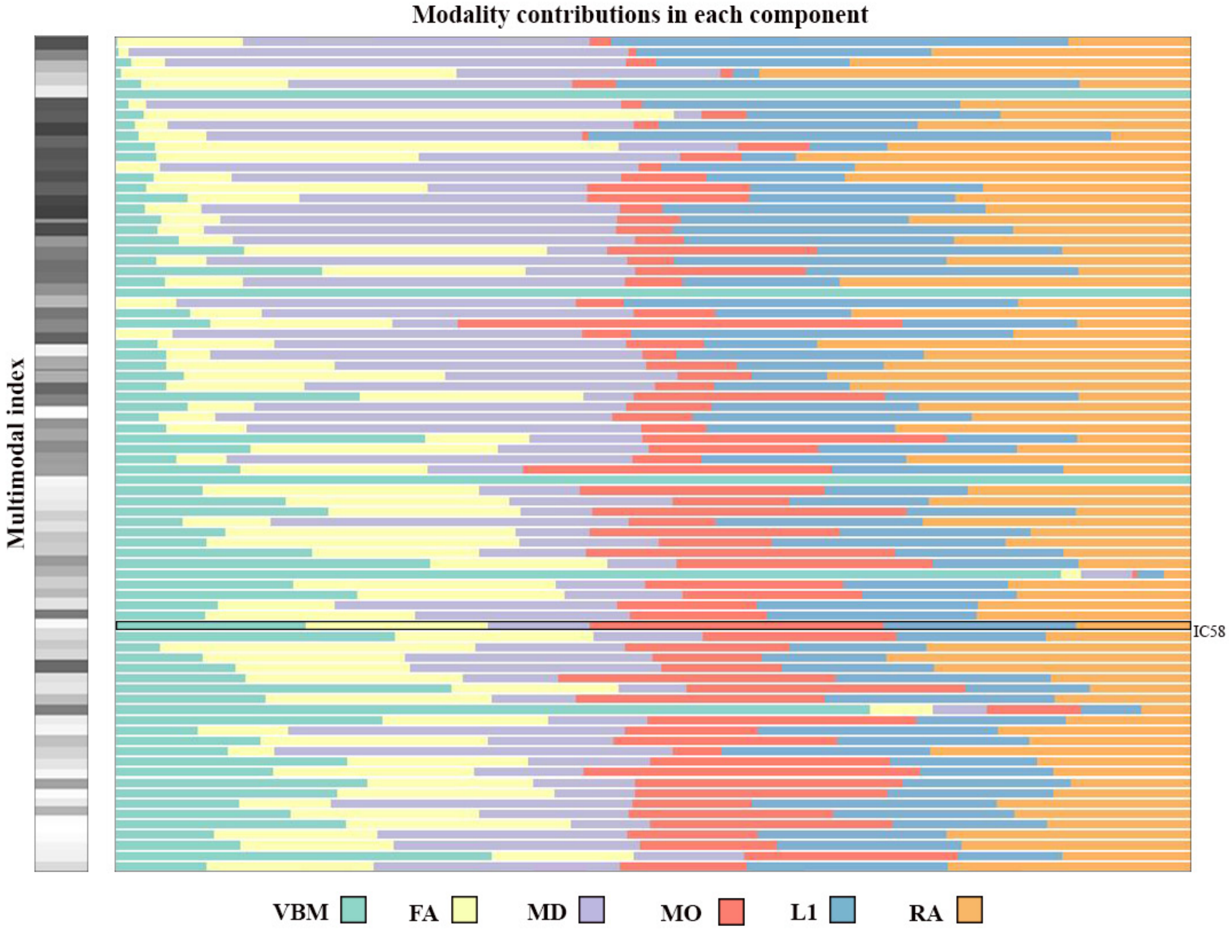

Figure S2 Modality contributions and multimodal index of each component (80 components). MMI showing left demonstrates the degree of multimodality (equation S1), i.e., the closer coded color to dark gray means the more dominant one modality in the components contributes, and in contrast the closer coded color to bright white shows the more equally each modality in the components contributes. MO, mode of anisotropy; RA, radial diffusivity; MD, mean diffusivity; FA, fractional anisotropy; L1, axial diffusivity; VBM, voxel-based morphometry.

6. Spatial distribution of VBM and DTI measures in the component with significant group effect

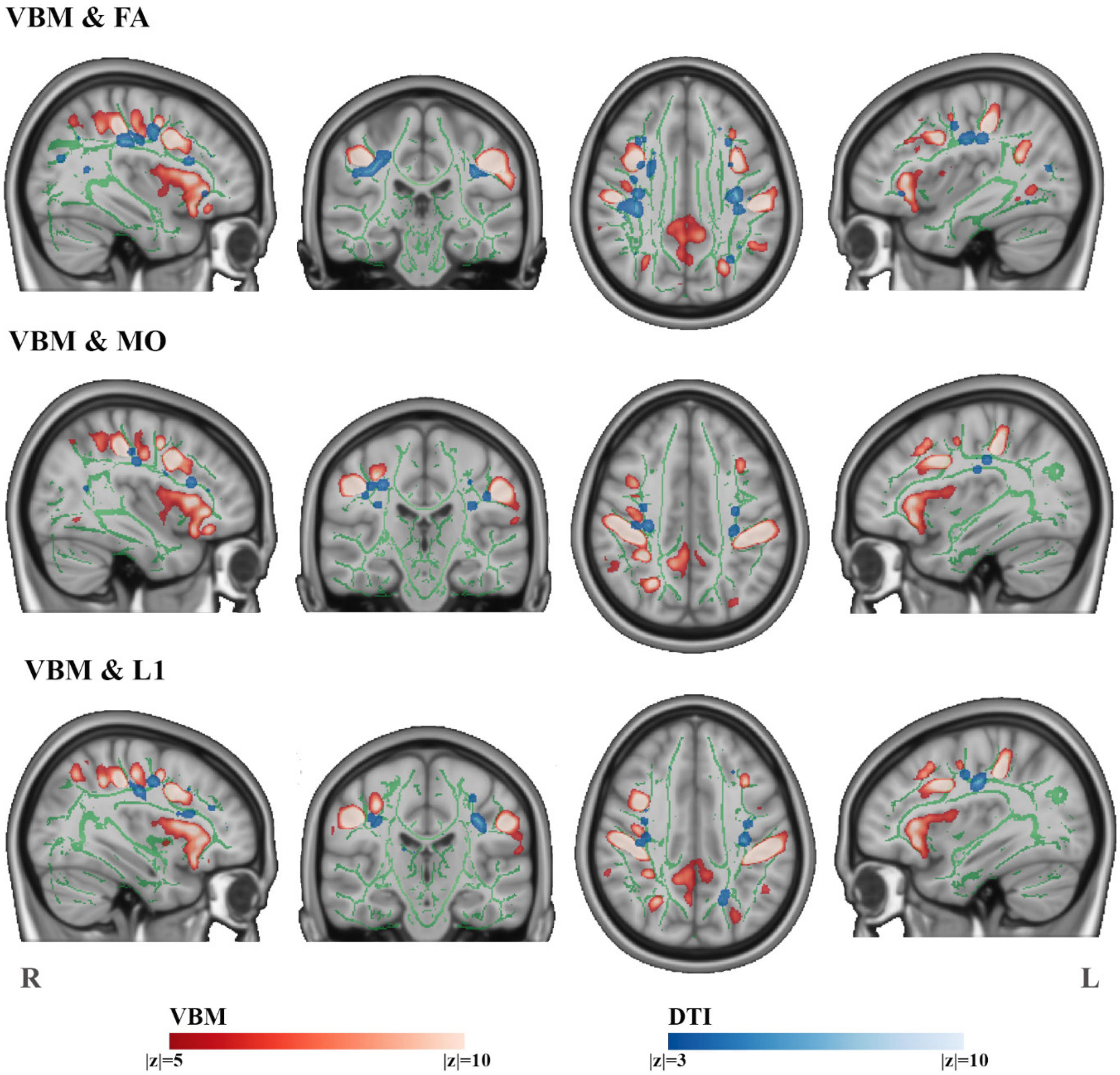

Figure S3 Spatial distribution of VBM and DTI measures in the component with significant group effect (IC58). The figure displays the top-three loading DTI measures in the component overlaying with the VBM spatial map, which indicates autism-relating variations of gray matter volume and WM tracts that locate around frontal, pre-central and post-central areas are spatially interconnected. The VBM spatial maps are thresholded at $5<|Z|<10$, and the DTI spatial maps are thresholded at $3<|Z|<10$. VBM, voxel-based morphometry; DTI, diffusion tensor imaging; FA, fractional anisotropy; MO, mode of anisotropy; L1, axial diffusivity.

7. Sensitivity Analyses

All analyses were done using imputed data (except for Medication section). All analyses below controlled for the other confounders that we included in the main analyses.

1) IQ

It is important to consider the complicated effects of IQ on brain morphology and its potentially different effects in autism and control groups. Therefore, in addition to including IQ in the main model we checked the IQ-by-diagnosis interaction effect on the brain GM-WM covariation patterns. The main effect of diagnosis in IC58 was robust to inclusion of an IQ-by-diagnosis term (β=-0.193, t(336)=-3.614, p=3.473x10^{-4}). There was also a main effect of IQ (p=0.038) but no IQ-by-diagnosis effect (p=0.214).

2) Age

Age may have potential effects on gray and white matter morphology, therefore we did post-hoc analyses to check the effects of age and age-by-diagnosis interaction on the autism-related GW-WM covariation pattern (i.e., IC58) in our main analyses. We found there was a significant age effect on IC58 (β=-0.182, t(336)=-3.464, p=6.012x10^{-4}) but no significant age-by-diagnosis interaction (p=0.837), which demonstrated the participant loadings across the whole sample on this component would decrease with aging. After entering the age-by-group interaction effect in the GLM, the diagnostic group effect on IC58 was still significant (p=3.848x10-4). The age-related effects did not remarkably influence our case-control findings in the current study.

3) Sex

Similarly, sex may also have potential effects on the case-control difference that we found in the main analyses, we hence did post-hoc analyses to explore the effects of sex and sex-by-diagnosis interactions on IC58. Either sex (p=0.087) or sex-by-diagnosis interaction (p=0.782) has no significant effect on IC58. However, the group effect on IC58 was still significant in this post-hoc analysis (p=0.006).

4) Medication

There is no specific medication for autism. Medication is often prescribed for co-occurring psychiatric conditions (e.g., ADHD, anxiety, depression). Considering the potential effect of medication use on our findings, we additionally involved a categorical covariate indicating whether the individuals took psychotropic medication (acting on nervous system, Table S5) in the GLM. Medication data were available on 326 participants, of which 79 participants used psychotropic medications. After controlling for the medication use, IC58 still showed a significant group effect (β=-0.223, t(318)=-3.827 p=1.562×10^{-4}). Additionally, there was no significant association between medication use and IC58 (p=0.052). Investigating medication effects where medication use is so heterogeneous is complex.

Table S5. Information of the medication use and co-occurring psychiatric conditions in current sample.

**Medications**[a]		Autism (N)	Controls (N)
No medication use		94	116
Medication use[b]		67	12
	Antiepileptics	7	1
	Antipsychotic	18	1
	Psychostimulants and other drugs for ADHD	28	5
	Hypnotics and sedatives	20	0
	Other analgesics and antipyretics	1	3
	Antidepressant	18	5
	Antimigraine preparations	1	0
	Anxiolytics	1	0
Other medication use		15	22
Unknown/missing medication use		9	9
**Co-occurring psychiatric conditions**[a]		Autism (N)	Controls (N)
ADHD rating scales			
	with/without ADHD	81/86	18/116
	Unkown	18	25
DAWBA Anxiety[c]			
	with probablity>70%/≤50%	2/156	0/127
	Unknown	27	32
DAWBA Depression[c]			
	with probablity>70%/≤50%	3/139	1/111
	Unknown	43	47

[a] The reports are based on non-imputed data.

[b] There were 4 participants with autism and 1 participant without autism using 3 different psychotropic medications. There were 19 participants with autism and 1 participant without autism using 2 different psychotropic medications.

[c] There are no cut-offs to indicate participants with or without the conditions in DAWBA Anxiety and Depression scales, we hence summarized the participants into two categorical groups suggesting high and low risks of having the conditions.

5) Site

The participants in our study were recruited in parallel at three collaborating sites (London, Nijmegen and Mannheim). In the main analysis, we included scanner site as a covariate in the GLM. Here, we additionally checked the interaction effect of diagnosis and site to further evaluate the robustness of our autism-related findings. We calculated type-III analysis-of-deviance (likelihood-ratio chi-square) for the model including a site-by-diagnosis interaction term (component ~ group*site + age + sex + IQ).

No main effect of the site was found ($G^2(2)=4.884, p=0.087$). We found a modest significant site-by-diagnosis interaction ($G^2(2)=6.860, p=0.032$) which indicates our group effect was not entirely consistent across sites. The main group effect was partially diminished by the inclusion of the interaction term ($G^2(1)=2.917, p=0.088$). Upon visual inspection (Figure S4) we see that the diagnosis effect is quite evident across all sites. However, the Mannheim site (which had the smallest sample N=55) shows a truncated upper distribution in the control sample potentially reflected by the group-by-site analysis.

To further determine the robustness of our results to site effects we post-hoc analyzed case-control differences within each site separately while corrected for age, IQ and sex using GLM, similar to the main analyses. These analyses revealed case-control differences, consistent with the main findings (autism lower than control), in both the KCL ($\beta=-0.149$, $t(157)=-2.430$, $p=0.016$) and Nijmegen ($\beta=-0.337$, t(122)=-

3.121, p=0.002) samples. However Mannheim, results showed no case-control differences (β=0.015, t(50)=0.118, p=0.907). Additionally, we think that decomposing the sample by each site reduces the statistical power and representativeness of participants. Furthermore, LEAP implemented a stringent data collection pipeline (15), the data across sites is coherent and comparable. The statistically significant site-related effect may also attribute to the considerable heterogeneity of autism regarding, e.g., symptom severity.

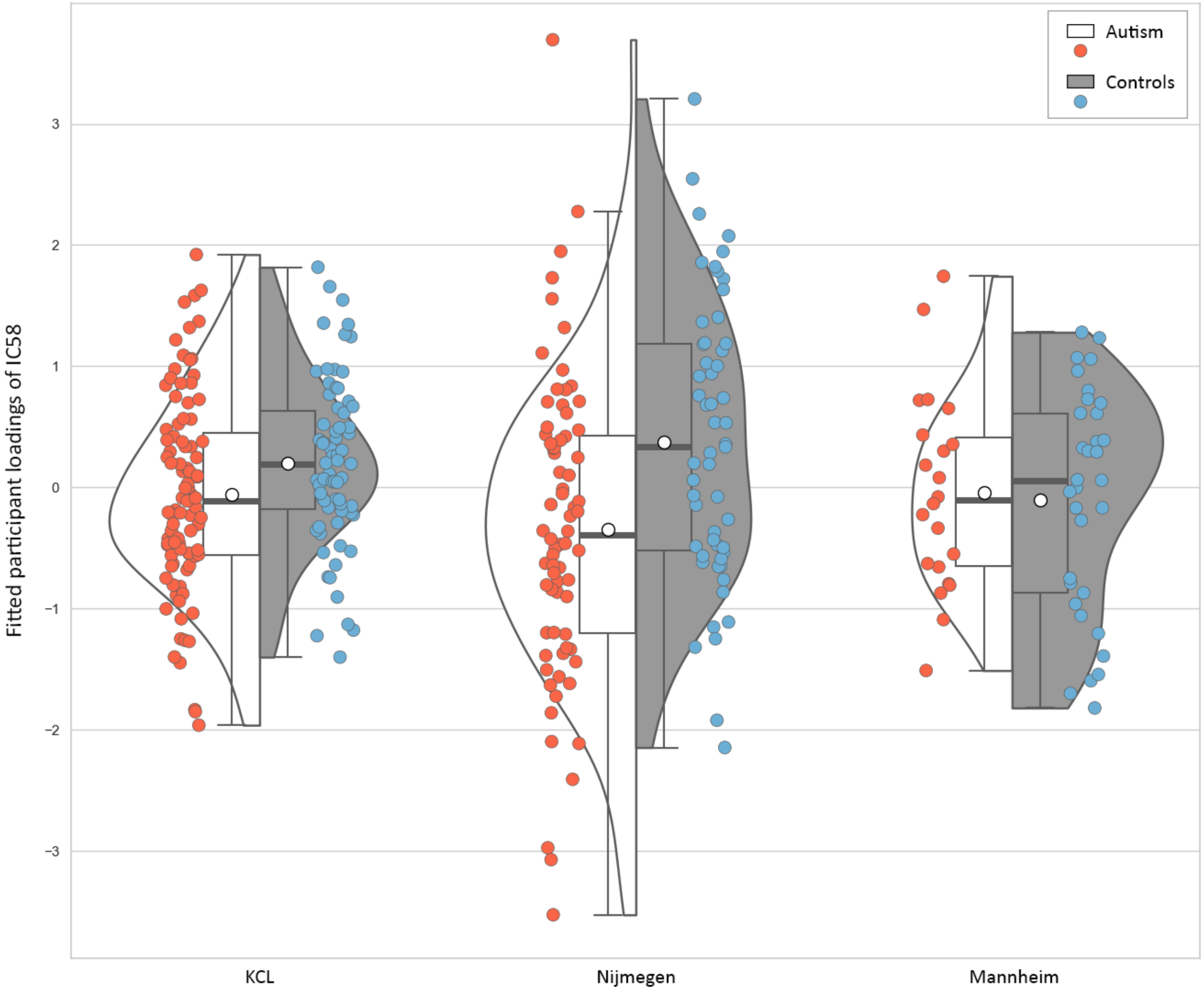

Figure S4 Distribution of the age-, sex-, and IQ corrected participant loadings of the component with significant group effect (IC58) in the main analyses across scanner sites. The white circle shows the mean of each group.

6) ADHD, anxiety and depression conditions

Concerning the potential effect of Attention Deficit Hyperactivity Disorder (ADHD), anxiety and depression co-occurrence, we separately included of the scores of ADHD DSM-5 rating scale (16), anxiety and depression from the Development and Well-Being Assessment (DAWBA) (17) as the additional covariates in the post-hoc analyses.

Considering the effects of co-occurring ADHD, we included the two dimensions (inattention and hyperactivity/impulsivity symptoms) of the ADHD rating scale as the additionally covariates. And the scale we used were based on parent report.

In addition to ADHD, we used the DAWBA anxiety and depression prediction scores to separately explore effects on group difference of GM-WM covariation pattern. In DAWBA, each scale reflects six levels of the possibility (i.e., from ~0.1% to >70%) to clinically meet the diagnostic criteria for anxiety or depression according to DSM-IV, DSM-5 and ICD10. The anxiety prediction score reflects the risk of having condition(s) in a group of anxiety disorders (obsessive-compulsive disorder (OCD), generalized anxiety, panic disorder, agoraphobia, PTSD, separation anxiety, social phobia, and specific phobia). The depression prediction score reflects the risk of having major depression. We used two dimensions of ADHD rating scales, and anxiety and depression imputed scores as additional covariates in the statistical model separately and treated DAWBA scores as continuous measures (details in Table S6).

The analysis showed that the group effect of IC58 was robust to the inclusion of continuous scores of inattention and hyperactivity/impulsivity symptoms (β=-0.181, t(335)=-3.114, p=0.002), and was robust to anxiety scores (β=-0.160, t(336)= -2.808, p=0.005) and depression scores (β=-0.186, t(336)=-3.462, $p=6.034 \times 10^{-4}$) as well. Additionally, there was no significant relation between inattention (p=0.983), hyperactivity/impulsivity (p=0.643), anxiety (p=0.134) or depression (p=0.442) scores and IC58.

Table S6. Sample characteristics of DAWBA anxiety and depression subscales

	Autism, N=185		Controls, N=159		Range
	Mean	SD	Mean	SD	
ADHD rating scale					
Inattentiveness	4.23	3.08	1.83	2.21	0~9
Hyperactivity/impulsivity	2.40	2.62	0.72	1.49	0~9
DAWBA					
Anxiety	1.914	1.120	1.191	0.661	0~5
Depression	0.710	1.087	0.443	0.867	0~5

8. All brain IC loadings of the significant CCA mode

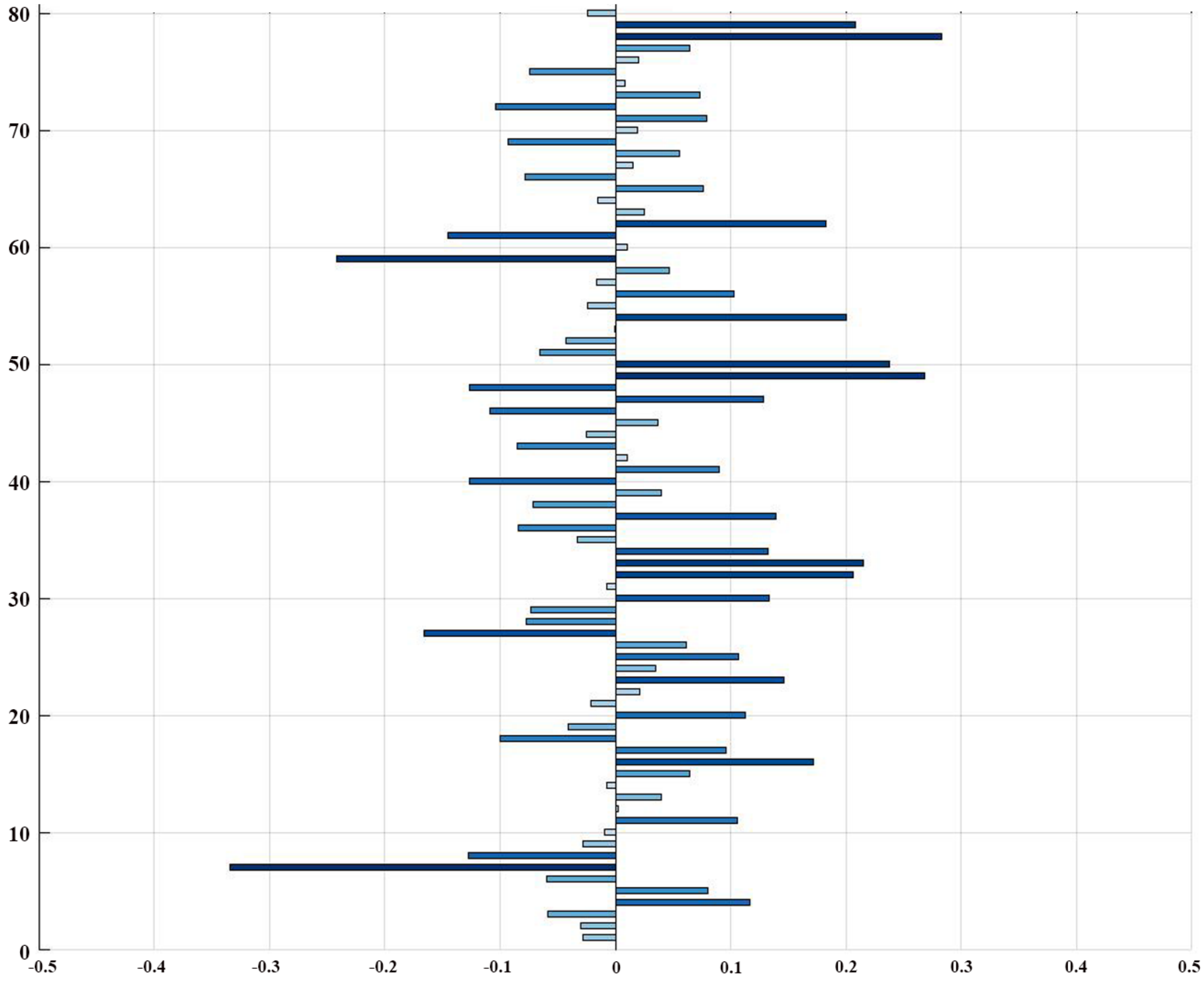

Figure S5. The loading of each component of the significant CCA mode in the main analysis

9. Reliability and stability assessment of CCA results

To assess the reliability of CCA results, we performed leave-one-subject-out (LOO) analysis leaving each participant out individually (185 folds, i.e., N=185). In each time, we individually correlated the CCA mode loadings of brain and behavior profiles, which were produced from LOO analysis of CCAs, with the corresponding loadings of the original (main) CCA analysis. Subsequently, the validity of the LOO analysis was determined by building a null distribution of the correlations between the randomized and original loadings of the CCA mode by separately randomizing the behavior and brain sets 1000 times. The means of correlation values larger than the 95th percentile of null distributions was considered as valid. The standard deviation (SD) of the correlations was used to reflect the stability of the CCA analysis and the loading of each variable. Consequently, the LOO analysis ($p<0.001$) indicated that the significant CCA mode of CCA analysis was reliably estimated. The SD of the estimator produced from LOO analysis and the results are shown in Table S7. The SDs were below 0.025 in the brain set and 0.048 in the behavioral set. The column 'SD/loading' shows the size of the SD with respect to the loading, i.e., how the difference of the loadings of LOO varies from the original loading. Therefore, a smaller value in this column reflects a more robust loading as assessed using LOO, for example, the maximum value of SD/loading for the top 10 high loading ICs (0.089) in the initial CCA is far less than the average (0.541), indicating the stability of the high loadings of CCA mode observed. The column 'loading/SD' exhibits the size of the loading in terms of the SD evaluated by LOO, contrary to SD/loading, a larger value in this column reflects a more robust loading, the other way around, the minimum value of loading/SD for the top 10 high loading ICs (11.208) in the initial CCA is far larger than the average (7.182). These measurements in the behavioral set showed a similar case of high loading variables.

Table S7. The information of the CCA mode loading estimators from leave-one-subject-out analysis

ICs	loading	SD	SD/loading	loading/SD	ICs	loading	SD	SD/loading	loading/SD
IC1	-0.028	0.002	0.084	11.900	IC45	0.037	0.017	0.444	2.252
IC2	-0.030	0.009	0.312	3.207	IC46	-0.109	0.010	0.093	10.746
IC3	-0.059	0.013	0.228	4.393	IC47	0.128	0.010	0.079	12.611
IC4	0.116	0.008	0.068	14.643	IC48	-0.126	0.012	0.096	10.420
IC5	0.081	0.006	0.074	13.578	IC49	0.268	0.024	0.089	11.208
IC6	-0.060	0.008	0.141	7.079	IC50	0.238	0.011	0.046	21.549
IC7	-0.334	0.018	0.054	18.467	IC51	-0.065	0.011	0.166	6.033
IC8	-0.127	0.012	0.094	10.589	IC52	-0.043	0.008	0.198	5.057
IC9	-0.029	0.010	0.335	2.984	IC53	-0.001	0.009	16.716	0.060
IC10	-0.009	0.008	0.879	1.137	IC54	0.200	0.012	0.061	16.523
IC11	0.106	0.012	0.112	8.916	IC55	-0.024	0.010	0.401	2.496
IC12	0.002	0.011	5.569	0.180	IC56	0.103	0.022	0.216	4.640
IC13	0.040	0.013	0.322	3.102	IC57	-0.017	0.013	0.795	1.259
IC14	-0.007	0.006	0.860	1.163	IC58	0.047	0.011	0.234	4.277
IC15	0.065	0.012	0.182	5.504	IC59	-0.242	0.014	0.060	16.779
IC16	0.172	0.011	0.064	15.743	IC60	0.010	0.008	0.754	1.327
IC17	0.096	0.012	0.125	7.974	IC61	-0.145	0.014	0.098	10.159
IC18	-0.099	0.012	0.116	8.635	IC62	0.183	0.014	0.079	12.667
IC19	-0.040	0.012	0.296	3.377	IC63	0.025	0.011	0.428	2.335
IC20	0.112	0.012	0.102	9.759	IC64	-0.015	0.008	0.562	1.780
IC21	-0.021	0.010	0.487	2.055	IC65	0.076	0.011	0.140	7.141
IC22	0.021	0.015	0.733	1.365	IC66	-0.078	0.010	0.125	7.994

IC23	0.146	0.013	0.090	11.086	IC67	0.016	0.006	0.366	2.732
IC24	0.035	0.011	0.312	3.206	IC68	0.056	0.017	0.300	3.333
IC25	0.107	0.013	0.122	8.208	IC69	-0.093	0.018	0.196	5.094
IC26	0.062	0.010	0.169	5.916	IC70	0.019	0.010	0.539	1.855
IC27	-0.165	0.011	0.067	14.947	IC71	0.079	0.010	0.133	7.541
IC28	-0.078	0.010	0.130	7.666	IC72	-0.104	0.025	0.242	4.135
IC29	-0.073	0.014	0.189	5.289	IC73	0.073	0.018	0.249	4.014
IC30	0.134	0.015	0.110	9.110	IC74	0.009	0.010	1.130	0.885
IC31	-0.007	0.011	1.599	0.625	IC75	-0.075	0.011	0.143	7.006
IC32	0.206	0.012	0.060	16.772	IC76	0.020	0.014	0.670	1.493
IC33	0.215	0.014	0.063	15.779	IC77	0.064	0.012	0.185	5.413
IC34	0.132	0.015	0.113	8.833	IC78	0.283	0.016	0.055	18.153
IC35	-0.033	0.009	0.285	3.505	IC79	0.208	0.013	0.062	16.096
IC36	-0.084	0.012	0.141	7.076	IC80	-0.024	0.008	0.313	0.352
IC37	0.139	0.018	0.127	7.892	ADI Social	0.148	0.047	0.316	3.161
IC38	-0.072	0.012	0.169	5.921	ADI Comm.	0.086	0.025	0.292	3.420
IC39	0.040	0.010	0.262	3.822	ADI RRB	0.449	0.035	0.079	12.696
IC40	-0.126	0.008	0.067	15.028	ADOS SA	0.331	0.044	0.133	7.523
IC41	0.090	0.016	0.182	5.480	ADOS RRB	-0.432	0.026	0.060	16.628

IC42	0.010	0.008	0.777	1.286	SRS	-0.253	0.031	0.121	8.281
IC43	-0.085	0.010	0.115	8.717	RBS	-0.065	0.023	0.355	2.815
IC44	-0.025	0.010	0.417	2.397	SSP	0.365	0.048	0.130	7.669

SD, standard deviation; IC, independent component.

10. Probing potential effect of the imputed SSP scores on CCA outputs

As the SSP scale utilized in analysis was highly imputed (42%) we repeated our CCA excluding the SSP scores. The output of the CCA without SSP did not differ greatly from the CCA of all measures. Therefore, the inclusion of the SSP data is warranted in the current study as sensory processing is of core importance in autism and the imputation performance was good (18).

We found a significant multivariate correlation between brain components and the 7 remaining behavioral scales (r=0.815, corrected p=0.017, Figure S6). In this multivariate associated pattern, covariation of brain areas that showed the strong contributions to the correlation with autism symptoms are same to the initial CCA, in which IC7 mainly included right inferior longitudinal fasciculus (ILF), inferior fronto-occipital fasciculus (IFOF), corticospinal tract (CST), and IC78 mainly involved bilateral anterior thalamic radiation and superior longitudinal fasciculus (SLF). In the brain components, IC7 and IC78 constantly exhibited strong contributions to the correlation with and without SSP. From a phenotypic perspective the loadings of ADI RRB, ADOS SA and ADOS RRB subscales were similar in both CCAs.

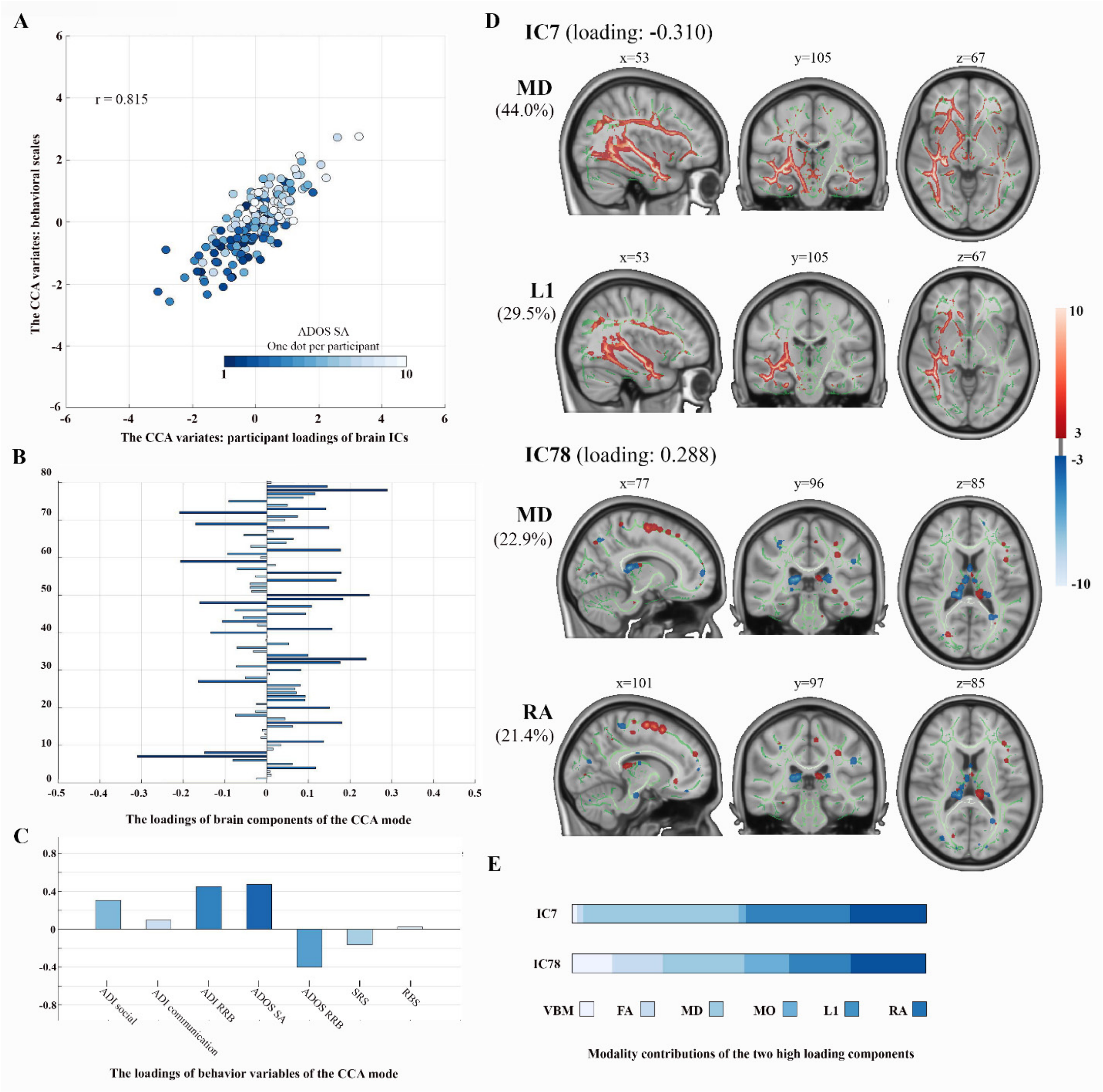

Figure S6 The multivariate association pattern (i.e., CCA mode) was found significant between the two sets of brain components and 7 behavioral profiles (without SSP). A displays the scatterplot of this correlation (between the CCA mode), and x, y axes are the pair of CCA variates. One dot in each participant is coded with gradient color regarding to the SA subscale of ADOS. B demonstrates the loading of each brain component in this CCA mode. C shows the loading of each behavioral subscale in this CCA mode. D exhibits the two brain components with the strong contribution to the correlation with autism symptoms,

where the top two loading modalities in each component are shown in the figure. The canonical loading of each component is shown in the brackets. The modality spatial maps are thresholded at $3<|Z|<10$. E shows the modality contributions to the brain components displayed in D. The CCA was only performed in autism group. CCA, canonical correlation analysis; ADI, Autism Diagnostic Interview-Revised; ADOS, Autism Diagnostic Observational Schedule 2; SA, social affect; RRB, restricted repetitive behavior; SRS, Social Responsiveness Scale 2nd Edition; RBS, Repetitive Behavior Scale-Revised; SSP, Short Sensory Profile; IC, independent component; MO, mode of anisotropy; RA, radial diffusivity; MD, mean diffusivity; FA, fractional anisotropy; L1, axial diffusivity; VBM, voxel-based morphometry.

11. Additional exploratory analyses with respect to IQ

Since IQ is linked to brain morphology and behavioral traits of autism, including IQ as a covariate may reduce the sensitivity to explore the relations of interest. To determine if this was occurring, we subsequently re-ran the models excluding IQ as a covariate. When IQ was excluded as a covariate in the GLM model, IC58 (the component significantly related to autism in main analyses) gave similar results although it did not survive FDR correction exploring case-control differences (β=-0.182, t(338)=-3.422, p=6.990x10^{-4}, adjusted p=0.056), and similarly we did not find any significant univariate brain-behavior associations (FDR corrected p>0.190).

We checked the associations between brain ICs and IQ using the GLM model at last. The results demonstrated that there were 6 ICs (IC6, IC27, IC36, IC40, IC52, IC57) significantly related to IQ after FDR (FDR corrected p<0.02). The autism-related IC found in the main analyses (IC58) was not significantly associated with IQ after FDR correction.

Supplementary References

1. Mei T, Llera A, Floris DL, Forde NJ, Tillmann J, Durston S, et al. Gray matter covariations and core symptoms of autism: the EU-AIMS Longitudinal European Autism Project. Mol Autism. 2020;11(1):86.

2. Bastiani M, Cottaar M, Fitzgibbon SP, Suri S, Alfaro-Almagro F, Sotiropoulos SN, et al. Automated quality control for within and between studies diffusion MRI data using a non-parametric framework for movement and distortion correction. Neuroimage. 2019;184:801-12.

3. Perrone D, Aelterman J, Pizurica A, Jeurissen B, Philips W, Leemans A. The effect of Gibbs ringing artifacts on measures derived from diffusion MRI. Neuroimage. 2015;120:441-55.

4. Sotiropoulos SN, Moeller S, Jbabdi S, Xu J, Andersson JL, Auerbach EJ, et al. Effects of image reconstruction on fiber orientation mapping from multichannel diffusion MRI: reducing the noise floor using SENSE. Magn Reson Med. 2013;70(6):1682-9.

5. Koay CG, Basser PJ. Analytically exact correction scheme for signal extraction from noisy magnitude MR signals. J Magn Reson. 2006;179(2):317-22.

6. Rutter M, Le Couteur A, Lord C. Autism Diagnostic Interview-Revised. Los Angeles: Western Psychological Services; 2003.

7. Lord C, Rutter M, DiLavore PC, Risi S, Gotham K, Bishop S. Autism DiagnosticObservation Schedule, Second Edition (ADOS-2) manual (part I): modules 1–4. Torrance: Western Psychological Services; 2012.

8. Constantino JN, Gruber CP. Social Responsiveness Scale. 2nd ed. Los Angeles: Western Psychological Services; 2012.

9. Bodfish JW, Symons FJ, Parker DE, Lewis MH. Varieties of repetitive behavior in autism: comparisons to mental retardation. J Autism Dev Disord. 2000;30(3):237-43.

10. Tomchek SD, Dunn W. Sensory processing in children with and without autism: a comparative study using the short sensory profile. Am J Occup Ther. 2007;61(2):190-200.

11. Hotelling H. Relations between two sets of variates. Biometrika. 1936;28:312-77.

12. Winkler AM, Renaud O, Smith SM, Nichols TE. Permutation inference for canonical correlation analysis. Neuroimage. 2020;220:117065.

13. Haufe S, Meinecke F, Gorgen K, Dahne S, Haynes JD, Blankertz B, et al. On the interpretation of weight vectors of linear models in multivariate neuroimaging. Neuroimage. 2014;87:96-110.

14. Francx W, Llera A, Mennes M, Zwiers MP, Faraone SV, Oosterlaan J, et al. Integrated analysis of gray and white matter alterations in attention-deficit/hyperactivity disorder. Neuroimage Clin. 2016;11:357-67.

15. Loth E, Charman T, Mason L, Tillmann J, Jones EJH, Wooldridge C, et al. The EU-AIMS Longitudinal European Autism Project (LEAP): design and methodologies to identify and validate stratification biomarkers for autism spectrum disorders. Mol Autism. 2017;8:24.
16. DuPaul GJ, Power TJ, Anastopoulos AD, Reid R. ADHD Rating Scale—5 for Children and Adolescents Checklists, Norms, and Clinical Interpretation. New York: Guilford Publications; 2016.
17. Goodman R, Ford T, Richards H, Gatward R, Meltzer H. The Development and Well-Being Assessment: description and initial validation of an integrated assessment of child and adolescent psychopathology. J Child Psychol Psychiatry. 2000;41(5):645-55.
18. Llera A, Brammer M, Oakley B, Tillmann J, Zabihi M, Mei T, et al. Evaluation of data imputation strategies in complex, deeply-phenotyped data sets: the case of the EU-AIMS Longitudinal European Autism Project. arXiv pre-print server. 2022.
